# Intranasal analgesia for acute moderate to severe pain in children – a systematic review and meta-analysis

**DOI:** 10.1186/s12887-023-04203-x

**Published:** 2023-08-18

**Authors:** Marcus Glenton Prescott, Ekaterina Iakovleva, Melanie Rae Simpson, Sindre Andre Pedersen, Daniel Munblit, Odd Martin Vallersnes, Bjarne Austad

**Affiliations:** 1 Department of Public Health and Nursing, Faculty of Medicine and Health Sciences, Norwegian University of Science and Technology (NTNU), Trondheim, Norway; 2 Emergency Department, St. Olavs Hospital, Trondheim, Norway; 3 Trondheim Municipal Out of Hours Primary Care Service, Trondheim, Norway; 4 Department of Pediatrics and Pediatric Infectious Diseases, Institute of Child´s Health, Sechenov First Moscow State Medical University (Sechenov University), Moscow, Russia; 5 Library Section for Medical and Health Sciences, NTNU University Library, Norwegian University of Science and Technology (NTNU), Trondheim, Norway; 6 Florence Nightingale Faculty of Nursing, Midwifery and Palliative Care, Care for Long Term Conditions Division, King´s College London, London, UK; 7 Department of General Practice, University of Oslo, Oslo, Norway; 8 Oslo Municipal Out of Hours Primary Care Service, Oslo, Norway; 9 Øya Medical Center, Trondheim, Norway

**Keywords:** Intranasal, Nasal, Analgesia, Pain, pediatric, Paediatric, Fentanyl, Ketamine, Dihydromorphine, Diclofenac

## Abstract

**Background:**

Children in acute pain often receive inadequate pain relief, partly from difficulties administering injectable analgesics. A rapid-acting, intranasal (IN) analgesic may be an alternative to other parenteral routes of administration. Our review compares the efficacy, safety, and acceptability of intranasal analgesia to intravenous (IV) and intramuscular (IM) administration; and to compare different intranasal agents.

**Methods:**

We searched Cochrane Library, MEDLINE/PubMed, Embase, Web of Knowledge, Clinicaltrials.gov, Controlled-trials.com/mrcr, Clinicaltrialsregister.eu, Apps.who.int/trialsearch. We also screened reference lists of included trials and relevant systematic reviews. Studies in English from any year were included.

Two authors independently assessed all studies. We included randomised trials (RCTs) of children 0–16, with moderate to severe pain; comparing intranasal analgesia to intravenous or intramuscular analgesia, or to other intranasal agents. We excluded studies of procedural sedation or analgesia.

We extracted study characteristics and outcome data and assessed risk of bias with the ROB 2.0-tool. We conducted meta-analysis and narrative review, evaluating the certainty of evidence using GRADE.

Outcomes included pain reduction, adverse events, acceptability, rescue medication, ease of and time to administration.

**Results:**

We included 12 RCTs with a total of 1163 children aged 3 to 20, most below 10 years old, with a variety of conditions. Our review shows that:

- There may be little or no difference in pain relief (single dose IN vs IV fentanyl MD 4 mm, 95% CI -8 to 16 at 30 min by 100 mm VAS; multiple doses IN vs IV fentanyl MD 0, 95%CI -0.35 to 0.35 at 15 min by Hannallah score; single dose IN vs IV ketorolac MD 0.8, 95% CI -0.4 to 1.9 by Faces Pain Scale-Revised), adverse events (single dose IN vs IV fentanyl RR 3.09, 95% CI 0.34 to 28.28; multiple doses IN vs IV fentanyl RR 1.50, 95%CI 0.29 to 7.81); single dose IN vs IV ketorolac RR 0.716, 95% CI 0.23 to 2.26), or acceptability (single dose IN vs IV ketorolac RR 0.83, 95% CI 0.66 to 1.04) between intranasal and intravenous analgesia (low certainty evidence).

- Intranasal diamorphine or fentanyl probably give similar pain relief to intramuscular morphine (narrative review), and are probably more acceptable (RR 1.60, 95% CI 1.42 to 1.81) and tolerated better (RR 0.061, 95% CI 0.03 to 0.13 for uncooperative/negative reaction) (moderate certainty); adverse events may be similar (narrative review) (low certainty).

- Intranasal ketamine gives similar pain relief to intranasal fentanyl (SMD 0.05, 95% CI -0.20 to 0.29 at 30 min), while having a higher risk of light sedation (RR 1.74, 95% CI 1.30 to 2.35) and mild side effects (RR 2.16, 95% CI 1.72 to 2.71) (high certainty). Need for rescue analgesia is probably similar (RR 0.85, 95% CI 0.62 to 1.17) (moderate certainty), and acceptability may be similar (RR 1.15, 95% CI 0.89 to 1.48) (low certainty).

**Conclusions:**

Our review suggests that intranasal analgesics are probably a good alternative to intramuscular analgesics in children with acute moderate to severe pain; and may be an alternative to intravenous administration. Intranasal ketamine gives similar pain relief to fentanyl, but causes more sedation, which should inform the choice of intranasal agent.

**Supplementary Information:**

The online version contains supplementary material available at 10.1186/s12887-023-04203-x.

## Background

Acute pain is a common presenting complaint in children [1–3], and may be the chief complaint in one third of paediatric patients in emergency rooms [4]. Common causes include acute abdomen, injuries, and migraines. Although causes differ between countries, acute pain in children remains an issue worldwide [5–8]. Despite this, children are at increased risk of inadequate pain relief [1, 9, 10], possibly because of difficulties in establishing intravenous (IV) or intramuscular (IM) access, or uncertainty in choice of medications or dosages [1, 11–13].

Intranasal (IN) administration is an alternative to injectables [14]. Here medication is administered by adding a nebulizer-tip to a syringe, or by made-for-purpose formulations in standard dose syringes [14]. Utilising the vascular plexus of the nasal mucosa, intranasal administration gives rapid drug absorption, and avoids first pass metabolism [15–17]. Intranasal analgesia may be faster and easier to administer than intravenous/intramuscular, and less painful and distressing for children and caregivers. Furthermore, severe adverse events of intranasal opioids such as respiratory depression or sedation can be reversed by intranasal administration of naloxone [18, 19].

A 2014 systematic review found two relevant randomised clinical trials (RCTs), comparing intranasal fentanyl to intravenous and intramuscular morphine; but did not compare other agents or different intranasal agents to each other [20]. A 2020 systematic review compared intranasal ketamine to fentanyl, finding them equivalent [21]. A review comparing intranasal agents to each other, and intranasal to other routes, is however lacking. Furthermore, additional trials have been conducted since these reviews were published.

The aim of this systematic review was to compare the efficacy, safety, and acceptability of intranasal analgesia to intravenous and intramuscular administration in the treatment of acute pain in children and to compare different intranasal agents.

## Methods

The study protocol was registered in PROSPERO prior to starting the review, ID CRD42021238232. This systematic review was conducted in accordance with the MECIR guidelines as described in the Cochrane Handbook for Systematic Reviews of Interventions 2^nd^ edition [22].

Eligibility criteria.

We included RCTs in English, without restrictions on publication year or status; with children aged 0–16, with acute moderate to severe pain (equivalent to Visual Analogue Scale (VAS) score of ≥ 6/10 [23]) from any cause, in any setting; receiving intranasal analgesia compared to another intranasal agent or another route of administration; measuring at least one of our primary outcomes:

- pain at baseline, pain reduction at all time points as measured by validated pain score
- adverse events (including sedation)
- rescue medication

Secondary outcomes include satisfaction and acceptability, time to and ease of administration. We excluded studies of procedural analgesia or sedation.

### Search methods

We searched The Cochrane Central Register of Controlled Trials (CENTRAL), MEDLINE/PubMed, Embase, Web of Knowledge, ClinicalTrials.gov, Controlled-trials.com/mrcr, Clinicaltrialsregister.eu, Apps.who.int/trialsearch. The searches were last updated May 12^th^ 2022, with an additional search in MEDLINE and Embase September 7^th^ 2022. An updated search in Embase was conducted ahead of publication June 19^th^ 2023. See Additional file 1 for search strategies. Reference lists of included trials and relevant systematic reviews were also screened for additional eligible trials.

### Data collection and analysis

Using the Covidence-tool [24], two review authors (MGP, EI) independently screened the title and/or abstract of every record and investigated potentially relevant articles in full text. Two authors (MGP, EI) independently extracted key study and outcome data in a standardised data extraction form. Protocols for all included studies were identified when possible, and authors were contacted directly for clarifications or to request missing data in published reports. Disagreements were resolved by discussion or by including a third author (MRS). Where questions arose about study relevance, other reviewers were consulted (BA, OMV, DM).

Two authors (MPG, EI) independently assessed the risk of bias of each study using the ROB 2.0 [25]. Disagreements were resolved by discussion or by consultation with a third author (MRS, DM).

All review authors independently assessed the certainty of the evidence (high, moderate, low, or very low) using the GRADE tool [26]. Disagreements were resolved by discussion.

### Data synthesis

We performed standard pairwise meta-analyses using a random effects model in Stata for each treatment comparison with at least two eligible studies [27]. Other data were presented in a narrative form.

Sensitivity analysis was carried out to assess inclusion/exclusion of Graudins 2015 on pain scores (converting median differences to mean differences (MDs) with 95% confidence intervals (95%CI)) and Reynolds 2018 (combining adverse events drowsiness and sleepiness to represent sedation) (Additional files 2 and 3).

Dichotomous data was expressed as absolute risk or risk ratios with 95%CIs, continuous data as MDs and/or standardised mean differences (SMDs) with 95% CIs and/or standard deviations (SDs). Where possible, pain scores were converted to a common 100 mm VAS. Mean differences reported by 100 mm VAS were compared to the definitions of minimal (10 mm), appreciable (20 mm and 30 mm), and substantial (50 mm) differences in pain improvement [28]. Sedation scales, use of rescue medication and acceptability/satisfaction/tolerability were dichotomised.

In cases of clinical, methodological, or statistical heterogeneity, applicability and significance of the heterogeneity was discussed within the group. Statistical heterogeneity was identified by visual identification of forest plots and by using a standard Chi2-test, with a significance level of alpha = 0.1. Heterogeneity for meta-analysed studies was examined by the I2 statistic. An I2-statistic in the range 0–40% may not indicate a significant or important level of inconsistency [22]. Where found, this heterogeneity was discussed in the team, and potential reasons determined.

## Results

### Study selection

**Fig. 1:**
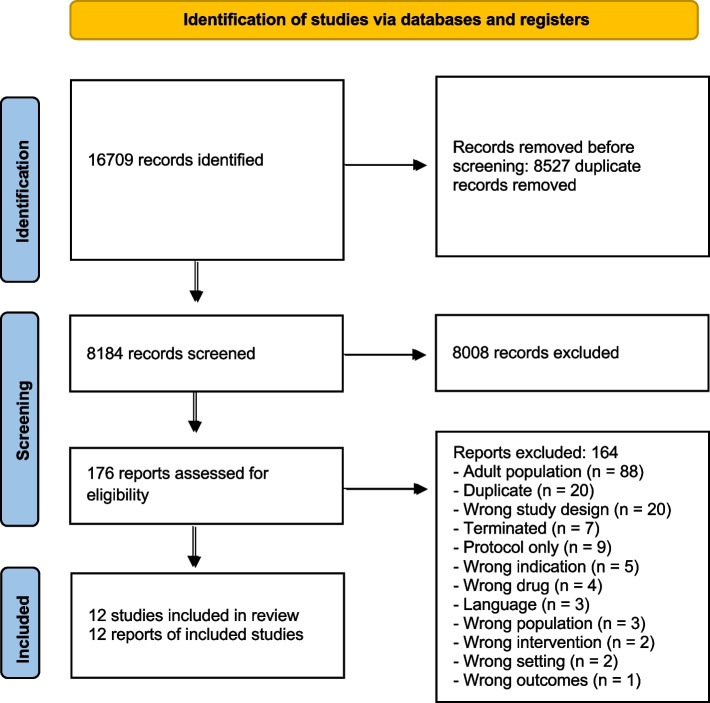
PRISMA flow diagram

We included twelve trials in the review. See Fig. 1: PRISMA flow diagram.

### Study characteristics

The twelve trials had a total of 1163 participants. Three trials (*n* = 158) compared intranasal analgesia to intravenous administration [29–31]. Three trials (*n* = 518) compared intranasal analgesia to intramuscular analgesia [32–34]. Six trials (*n* = 487) compared different intranasal agents (35. 36, 37, 38, 39, 40).

The six trials comparing routes of administration included children aged 2–20, though only 2 had participants over 15 [31, 34]. All trials except Tsze 2022, had mean or median ages under 11. Cause of pain was fractures in four studies [29, 32–34], post-operative in one [30]; and migraine headache in one [31]. Most studies looked only at single dose regimens [31, 33, 34], with 2 giving new doses every 5 min up to a certain limit [29, 30]. Furthermore, dosages varied, with IN fentanyl given at 0.5mcg/kg [30], 1mcg/kg [33], or 1.4mcg/kg [29]; while doses of IN diamorphine at 0.1 mg/kg, and IM morphine at 0.2 mg/kg [32–34] were the same in all studies. IV morphine at 0.1 mg/kg [29], IV fentanyl at 0.5mcg/kg [30], IN ketorolac at 1.0 mg/kg and IV ketorolac at 0.5 mg/kg [31] were given in single studies.

Most studies used common pain scales, including versions of VAS/VNRS [29], FACES pain scale [34]; or both scales [31, 33, 33], using different scales for different ages. Only one study used the post-operative Hannallah scale [30].

All studies counted adverse events, though not always recording the same side effects, or for the same duration of time, ranging from 30 min [29, 32–34] to 24 h [31]. Rescue medication outside of protocol was recorded in four studies [29, 31, 33, 34] – note that 2 studies gave additional doses of the initial study drug.

Satisfaction, acceptability, and tolerance or reaction to treatment was recorded by different metrics in four studies [31, 33, 34].

Different intranasal agents were compared in children aged 3–17, all trials including children aged 8–13. Four studies included only patients with limb injuries or fractures [35–37, 39], while one included moderate to severe pain of extremities or abdomen [40]. Three studies gave only a single dose [36, 37, 40], one an additional dose at 20 min [39], and one gave additional doses as needed [35]. IN ketamine was given at 1 mg/kg [36, 39, 40] or 1.5 mg/kg [37]; while IN fentanyl was given at 1.5mcg/kg [35, 36, 39, 40] or 2.0mcg/kg [37, 38]. All except one of the studies measured pain by a variation over the FACES pain scale [35, 36, 39, 40], in addition one also used an 11pt NRS [40], the others using a 100 mm VAS. One study used 100 mm VAS alone [37]. Adverse events were recorded in all studies, though for different durations and with different definitions. Rescue medication was recorded in all studies. Satisfaction, acceptability, or tolerance were recorded in one study [36].

For further details on population, intervention, comparison, and outcomes, see Table 1: Characteristics of included studies.

**Table 1:** Characteristics of included studies

*Study*	*Population*	*Intervention*	*Comparison*	*Outcomes*
Studies comparing intranasal analgesia (IN) to intravenous (IV) administration				
Borland 2007 [29]	67 children, 7–15 years, 20-50 kg, with clinically deformed closed long-bone fractures	IN fentanyl 1.2mcg/kg, 150mcg/ml + IV NaCl (*n* = 33). First dose at 0 min, additional doses every 5 min until relief, refusal or maximal dose (2–4 doses, weight dependent). Blinded	IV morphine 0.1 mg/kg, 10 mg/ml + IN NaCl (*n* = 34). First dose at 0 min, additional doses every 5 min until relief, refusal or maximal dose (2–4 doses, weight dependent). Blinded	100 mm VAS at 0, 5, 10, 20, 30 min Adverse events for 30 min. Rescue medication. Verbal pain rating
Manjushree 2002 [30]	32 children, 4–8 years, after elective surgical procedures of 1 to 1.5 h, ASA I-II, Hannallah score > / = 4	IN fentanyl 0.5mcg/kg + IV NaCl (*n* = 16). Initial dose at 0 min, then every 5 min up to 30 min, pain relief or adverse events. Blinded	IV fentanyl 0.5mcg/kg + IN NaCl (*n* = 16). Initial dose at 0 min, then every 5 min up to 30 min, pain relief or adverse events. Blinded	Hannallah scale 10pt pain scores at 0, 5, 10, 15 min. Adverse events for 30 min. Dose requirements. Time to analgesia. Vitals
Tsze 2022 [31]	59 children, 8–17 years, with migraine headache, with moderate to severe pain	IN ketorolac 1 mg/kg, 30 mg/ml, + IV NaCl 0.9%, single dose (*n* = 29). Blinded	IV ketorolac, 30 mg/ml + IN NaCl 0,9%, single dose (*n* = 30). Blinded	Faces Pain Scale – Revised (FPS-R) at 0, 10, 30, 60, 120 min. Adverse events. Rescue medication. Tolerability
Studies comparing intranasal analgesia (IN) to intramuscular (IM) administration				
Kendall 2001 [32]	404 children, 3–16 years, with clinical fracture of a limb	IN diamorphine 0.1 mg/kg single dose (*n* = 207). Unblinded	IM morphine 0.2 mg/kg single dose (*n* = 209). Unblinded	Wong-Baker pain rating scale (WBFPS)/Visual analogue Scale (VAS) at 0, 5, 10, 20, 30 min, by parents, providers, and patients. Adverse events for 30 min. Acceptability for parents and providers. Patient prepared to have treatment again. Reaction to administration. Vitals
Wilson 1997 [34]	58 children, 4–17 years, with clinically diagnosed limb fracture	IN diamorphine 0.1 mg/kg (*n* = 30), single dose. Unblinded	IM morphine 0.2 mg/kg (*n* = 28), single dose. Unblinded	WBFPS/6pt VAS at 0, 5, 10, 20, 30 min. Parental acceptability. Adverse events for 30 min. Rescue analgesia
Younge 1999 [33]	47 children, 3–10 years, with clinical limb fracture	IN fentanyl 1.0mcg/kg (*n* = 24), 50mcg/ml, single dose. Unblinded	IM morphine 0.2 mg/kg (*n* = 23), 10 mg/ml, single dose. Unblinded	WBFPS 5pt pain score by patient and parents at 0, 5, 10, 20, 30 min. Tolerance 4pt score by parents at 0 min. Adverse events. Rescue analgesia. Vitals
Studies comparing intranasal analgesia (IN) agents				
Borland 2011 [35]	199 children, 7-15 years, with clinically deformed closed long-bone fractures	IN fentanyl 50mcg/ml, 1.5mcg/kg (*n* = 102). First dose at 0 min, additional doses as required. Blinded	IN fentanyl 300mcg/ml, 1.5mcg/kg (*n* = 97). First dose at 0 min, additional doses as required. Blinded	100 mm VAS or FPS-R at 0, 10, 20, 30 min. Adverse events for 30 min. Rescue analgesia. Vitals
Frey 2019 [37]	90 children, 8–17 years, with acute extremity injury, with moderate to severe pain at presentation	IN ketamine 1.5 mg/kg, 50 mg/ml, single dose, max. 100 mg (*n* = 45). 4/44 (9%) had received ibuprofen and 1 (2%) paracetamol before coming to the ED. Blinded	IN fentanyl 2mcg/kg, 50mcg/ml, single dose, max. 100mcg (*n* = 45). 4/42 (10%) had received ibuprofen and 2 (5%) paracetamol before coming to the ED. Blinded	100 mm VAS at 0, 15, 30, 60 min. Adverse events for 2 h, and at 30d. Rescue medication. Sedation, 5pt UMSS. Vital signs. Capnometry
Graudins 2015 [36]	80 children, 3–13 years, < 50 kg, with isolated limb injury and moderate to severe pain at presentation	IN ketamine 1 mg/kg, 100 mg/ml, single dose (*n* = 36). 33/36 (92%) also received ibuprofen. Blinded	IN fentanyl 1.5mcg/kg, 50mcg/ml, single dose (*n* = 37). 33/37 (89%) also received ibuprofen. Blinded	FPS-R/100mmVAS at 0, 15, 30, 60 min. Adverse events. Rescue medication. Satisfaction. Sedation, 5pt UMSS
Quinn 2021 [40]	22 children, 3–17 years, < 64 kg, with acute moderate to severe pain at presentation (extremity or abdominal)	IN ketamine 1 mg/kg, 100 mg/ml, single dose (n = 11). 2/11 (18%) had received ibuprofen and 2 (18%) paracetamol before the study drug. Blinded	IN fentanyl 1.5mcg/kg, 50mcg/ml, single dose (n = 11). Blinded. 2/11 (18%) had received ibuprofen and 2 (18%) paracetamol before the study drug. Blinded	NRS/WBFPS at 0, 10, 20, 30, 60 min. Adverse events. Sedation, 5pt University of Michigan Sedation Scale (UMSS). Rescue medication. Vitals
Reynolds 2017 [39]	91 children, 4–17 years, < 70 kg, with a suspected single-extremity fracture and moderate to severe pain at presentation	IN ketamine 1 mg/kg, 50 mg/ml, at 0 min (*n* = 46); 2^nd^ dose 0.5 mg/kg after 20 min as needed. Acetaminophen 15 mg/kg. 33/43 (79%) also received ibuprofen, 7 (16%) paracetamol, and 1 (2%) both. Blinded	IN fentanyl 1.5mcg/kg, 50mcg/ml, at 0 min (*n* = 45); 2^nd^ dose 0.75mcg/kg after 20 min as needed. 35/44 (80%) also received ibuprofen, 5 (11%) paracetamol, and 3 (7%) both. Blinded	FPS-R/100 mm VAS at 0, 10, 20, 30, 60 min. Adverse events for 60 min. Required 2nd dose. Additional rescue analgesia
Fein 2017[38]	49 children, 3–20 years, with Sickle Cell Disease with crisis	IN fentanyl 2mcg/kg, 50mcg/ml, single dose (*n* = 24). Max. 100mcg. Blinded. 1 (4%) had received paracetamol, 1 (4%) oxycodone, and 1 (4%) paracetamol + codeine within 2 h prior to study drug	IN NaCl 0.9%, single dose (*n* = 25). Blinded. 1 (4%) had received paracetamol + codeine, and 1 (4%) tramadol prior to study drug	WBFPS at 0, 10, 20, 30 min. Adverse events for 20 min. Hospitalisation, bouncebacks

### Risk of bias

**Fig. 2:**
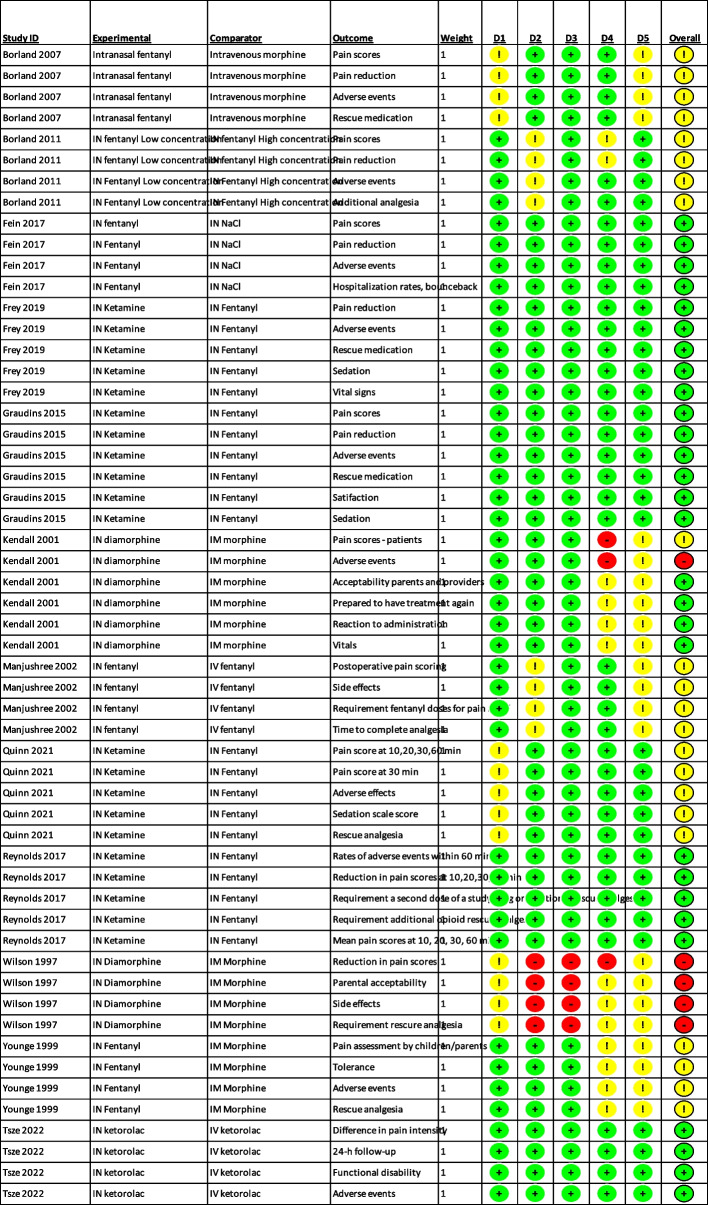
Summary of risk of bias

Two studies had outcomes with a high risk of bias – Wilson 1997 and Kendall 2001, both in part because of lack of blinding. We had some concerns for another five studies [29, 30, 33, 35, 40], while four studies were low risk for all outcomes [36–39]. The most common concerns were lack of adequate blinding and lack of a published protocol. See Fig. 2: Summary of risk of bias.

### Certainty of the evidence

For most comparisons, certainty of the evidence was low, usually because of relatively small numbers and single trials (Table 2: Summary of findings).

**Table 2:** Summary of findings

**Comparison 1a: Intranasal analgesia (IN) vs intravenous administration (IV):** Single dose IN fentanyl vs IV morphine in children aged 7–15 with acute fractures						
**Outcomes**	**Absolute effect (95% CI)**		**Relative effect**	**No of participants (studies)**	**Certainty of the evidence (GRADE)**	**What happens**
	**Risk with IV morphine**	**Risk with IN fentanyl**				
Pain 10 min	Mean pain score: 41 mm	Mean 5 mm higher (7 lower to 16 higher)		65 (1 RCT) [29]	Low^a^	Children receiving intranasal fentanyl may achieve similar reductions in pain scores at 10 and 30 min as those receiving intravenous morphine
Pain 30 min	Mean pain score: 33 mm	Mean 4 mm higher (8 lower to 16 higher)		65 (1 RCT) [29]	Low^a^	
Rescue medication	59 per 1000 children	61 per 1000 children (9 to 405)	RR 1.03 (0.15 – 6.89)	67 (1 RCT)^32^	Low^a^	
Mild adverse events	29 per 1,000 children	91 per 1,000 children (10 to 830)	RR 3.091 (0.338 to 28.281)	67 (1 RCT) [29]	Low^a,b^	
**Comparison 1b: Intranasal analgesia (IN) vs intravenous administration (IV):** Multiple doses IN fentanyl vs IV fentanyl in children aged 4–8 with postoperative pain						
**Outcomes**	**Absolute effect (95% CI)**		**Relative effect**	**No of participants (studies)**	**Certainty of the evidence (GRADE)**	**What happens**
	**Risk with IV fentanyl**	**Risk with IN fentanyl**				
Pain 10 min (Hannallah score)			MD -0.47 (-1.51 – 0.37)	32 (1 RCT)^33^	Low^a^	Patients receiving intranasal fentanyl may achieve similar reductions in pain scores at 10 and 15 min as those receiving intravenous fentanyl
Pain 15 min (Hannallah score)			MD 0 (-0.35 – 0.35)	32 (1 RCT)^33^	Low^a^	
Mild adverse events	125 per 1000 children	187.5 per 1000 children	RR 1.50 (0.29 – 7.81)	32 (1 RCT)^33^	Low^a^	
**Comparison 1c: Intranasal analgesia (IN) vs intravenous administration (IV):** Single dose IN ketorolac vs IV ketorolac in children aged migraine headache with migraine						
**Outcomes**	**Absolute effect (95% CI)**		**Relative effect**	**No of participants (studies)**	**Certainty of the evidence (GRADE)**	**What happens**
	**Risk with IV ketorolac**	**Risk with IN ketorolac**				
Pain 10 min			MD 0.9 (-0.4 – 2.2)	56 (1 RCT)^31^	Low^a^	Patients receiving intranasal ketorolac may achieve similar reductions in pain scores at 10 and 30 min as those receiving intravenous ketorolac
Pain 30 min			MD 0.8 (-0.4 – 1.9)	56 (1 RCT)^31^	Low^a^	
Rescue medication	170 of 1,000 children	220 per 1,000 children	RR 1.29 (0.44 – 3.73)	56 (1 RCT)^31^	Low^a^	
Mild adverse events	207 per 1,000 children	148 per 1,000 children	RR 0.716 (0.23 – 2.26)	56 (1 RCT)^31^	Low^a^	
Acceptability (child)	955 per 1,000 children	792 per 1,000 children	RR 0.83 (0.66 – 1.04)	46 (1 RCT)^31^	Low^a^	
a – single study, no published protocol b – underpowered to find differences in uncommon, but serious adverse events						
**Comparison 2: Intranasal analgesia (IN) vs intramuscular administration (IM)** IN fentanyl or IN diamorphine vs IN morphine in children aged 3–17 with acute moderate to severe pain						
**Outcomes**	**Absolute effect (95% CI)**		**Relative effect**	**No of participants (studies)**	**Certainty**	**What happens**
	**Risk with IM morphine**	**Risk with IN fentanyl/diamorphine**				
Pain 10 min	Kendall 2001 found lower mean pain scores in the IN diamorphine group than in the IM morphine group (MD 0.35, 95%CI -0.01, 0.71); Wilson 1997 found no difference in median pain scores between the IN diamorphine group and the IM morphine group (MD 0, 95%CI -0.24, 0.24) Younge 1999 found a 1-point lower median pain score in the IN fentanyl group than in the IM morphine group			490 (3 RCTs) [32–34]	Moderate^a^	Patients receiving intranasal fentanyl or diamorphine probably experience similar or more pain reduction at 10 and 30 min than those receiving intramuscular morphine. Any difference is unlikely to be clinically relevant
Pain 30 min	Kendall 2001 found lower mean pain scores in the IN diamorphine group than in the IM morphine group (MD 0.43, 95%CI 0.08, 0.78) Wilson 1997 found no difference in median pain scores between the IN diamorphine group and the IM morphine group (MD 0, 95% CI -0.55, 0.55) Younge 1999 found no difference in median pain score between the IN fentanyl group and the IM morphine group			483 (3 RCTs) [32–34]	Moderate^a^	
Rescue medication	45 per 1,000 children	50 per 1,000 children (23 – 108 children per 1,000)	RR 1.11 (0.51 – 2.40)	503 (3 RCTs) ^28–30^	Low^a,b^	Patients receiving intranasal fentanyl or diamorphine may experience similar risk of requiring rescue analgesia, as those receiving intramuscular morphine
Adverse events	Kendall 2001 reported total adverse events 49/203 (24%) in the IN diamorphine group, and 37/200 (18.5%) in the IM morphine group. All events were mild, except one case of vomiting in the IN diamorphine group; and over halt the events were local irritation at the site of administration. Neither Kendall 2001, Wilson 1997 or Younge 1999 found a difference in pulse, respiratory rate or GCS, or clinically significant in O2-saturations, but a high dropout rate in the IM morphine group			502 (3 RCTs) [32–34]	Low^a, b^	Patients receiving intranasal fentanyl or diamorphine may experience similar risk of adverse events, as those receiving intramuscular morphine
Acceptability (child): uncooperative / negative reaction	505 per 1,000 children	31 per 1,000 children (15 to 65 per 1,000 children)	RR 0.0611 (0.0291 to 0.1282)	449 (2 RCTs) [32, 34]	High^d^	Children are less likely to be uncooperative or have a negative reaction to intranasal analgesia than intramuscular morphine
Acceptability (child)	588 per 1,000 children	941 per 1,000 children (835 to 1,000 children)	RR 1.60 (1.42 to 1.81)	402 (1 RCT) [32]	Moderate^c^	Children probably find intranasal diamorphine more acceptable than intramuscular morphine
Acceptability (parents)	721 per 1,000 chilrden	966 per 1,000 children (887 to 1,000 children)	RR 1.34 (1.23 to 1.47)	389 (1 RCT) [32]	Moderate^c^	Parents probably find intranasal diamorphine more acceptable than intramuscular morphine
Acceptability (providers)	322 per 1,000 children	981 per 1,000 children (801 to 1,000 children)	RR 3.05 (2.49 to 3.73)	402 (1 RCT) [32]	High^d^	Providers find intranasal diamorphine more acceptable than intramuscular morphine
a – Wilson 1997 had high risk of bias, and some concerns in Kendall 2001 and Younge 1999 b – Imprecision: underpowered to find differences in uncommon events including rescue medication and adverse events c – Imprecision—single study d – Single study/only two studies, but upgraded because of large effect size						
**Comparison 3a: Comparison of different intranasal (IN) agents** IN ketamine vs IN fentanyl in children aged 3–17 with acute moderate to severe pain						
**Outcomes**	**Absolute effect (95% CI)**		**Relative effect**	**No of participants (studies)**	**Certainty**	**What happens**
	**Risk with IN fentanyl**	**Risk with IN ketamine**				
Pain 10–15 min			SMD 0.05 (-0.19 to 0.28)	263 (4 RCTs) [36, 37, 39, 40]	High	Patients receiving intranasal ketamine achieve similar pain reduction at 10–15 min, as those receiving intranasal fentanyl
Pain 30 min			SMD 0.05 (-0.20 to 0.29)	254 (4 RCTs) [36, 37, 39, 40]	High	Patients receiving intranasal ketamine achieve similar pain reduction at 30 min as those receiving intranasal fentanyl
Rescue medication	396 of 1,000 children	336 per 1,000 children (245 – 461 of 1,000 children)	RR 0.85 (0.62 – 1.17)	268 (4 RCTs) ^36–39^	Moderate^a^	Patients receiving intranasal ketamine probably experience a similar risk of requiring rescue analgesia as those receiving intranasal fentanyl
Mild adverse events	389 per 1,000 children	841 per 1,000 children (670 to 1,000 children)	RR 2.16 (1.72 to 2.71)	263 (4 RCTs) ^[[36, 37, 39, 40]]^	High	Patients receiving intranasal ketamine probably experience a higher risk of adverse events than those receiving intranasal fentanyl
Adverse events – Sedation	315 per 1,000 children	550 per 1,000 children (408 to 740 per 1000 children)	RR 1.74* (1.30 to 2.35)	261 (4 RCTs) ^[[36, 37, 39, 40]]^	High	Patients receiving intranasal ketamine experience a higher risk of sedation than those receiving intranasal fentanyl
Acceptability (child)	722 per 1,000 children	829 per 1,000 children (644 to 1,000 children)	RR 1.15 (0.89 to 1.48)	71 (1 RCT) [36]	Low^b^	Children may find intranasal ketamine equally acceptable to intranasal fentanyl
**Comparison 3b: Comparison of different intranasal (IN) agents** IN fentanyl vs IN placebo in children aged 3–20 with vaso-occlusive crisis of sickle cell disease						
**Outcomes**	**Absolute effect (95% CI)**		**Relative effect**	**No of participants (studies)**	**Certainty**	**What happens**
	**Risk with IN placebo**	**Risk with IN fentanyl**				
Pain 10 min	Mean pain reduction 1.0 (SD 2.0)	Mean pain reduction 2.2 (SD 2.6)		49 (1 RCT)^34^	Low^b^	Children receiving IN fentanyl may experience a 2 point greater reduction in pain than those receiving placebo at 10 min. This difference represents an appreciable change
Pain 30 min	Mean pain reduction 1.8 (SD 2.5)	Mean pain reduction 2.3 (SD 2.8)		49 (1 RCT)^34^	Low^b^	Children receiving IN fentanyl may experience no difference in reduction of pain compared to those receiving placebo at 30 min
Adverse events (in VOC)	There were no differences in serious adverse events. Two subjects in either group had hypotension that resolved spontaneously, and three in the intranasal fentanyl group had transient hypoxia			49 (1 RCT)^34^	Low^b^	There may be little or nor difference in adverse events between intranasal fentanyl and placebo in vaso-occlusive crises
**Comparison 3c: Comparison of different intranasal (IN) agents** Standard concentration IN fentanyl vs high concentration IN fentanyl in children aged 7–15 with fractures						
**Outcomes**	**Absolute effect (95% CI)**		**Relative effect**	**No of participants (studies)**	**Certainty**	**What happens**
	**Risk with high concentration IN fentanyl**	**Risk with standard concentration IN fentanyl**				
Pain 10 min	Median pain reduction 20 mm	Median pain reduction 20 mm	Median difference 0 (-5.2 – 5.2 mm)	189 (1 RCT)^35^	Low^b^	Patients receiving standard concentration (50mcg/ml) intranasal fentanyl may achieve similar pain relief at 10 min, as those receiving high concentration (300mcg/ml) intranasal fentanyl
Rescue medication	275 per 1,000 children	429 of 1,000 children (286 – 642 per 1,000 children)	RR 1.56 (1.04 – 2.34)	189 (1 RCT)^35^	Low^a^	Patients receiving standard concentration (50mcg/ml) intranasal fentanyl may experience a higher risk of requiring rescue analgesia than those receiving high concentration (300mcg/ml) intranasal fentanyl
Adverse events	320 per 1,000 children	225 per 1,000 children	RR 0.76 (0.47 – 1.23)	189 (1 RCT)^35^	Low^a^	Patients receiving standard concentration (50mcg/ml) intranasal fentanyl may experience similar risk of adverse events, as those receiving high concentration (300mcg/ml) intranasal fentanyl
a – Imprecision: underpowered to find differences in uncommon events, including rescue medication and serious adverse events b – Imprecision: single study						

Most comparisons had too few studies for formal statistical assessment of publication bias. A funnel plot comparing pain relief after intranasal ketamine or fentanyl did not lead to any suspicion of significant publication bias (Additional file 4). We noted multiple unfinished studies in clinical trial registries; many recorded as terminated because of low recruitment numbers, or a date of registration consistent with termination, though not explicitly stated (Additional file 5). This may indicate some risk of publication bias, though it seems unlikely to be significant.

### Effects of interventions

Studies for all comparisons measured pain at timepoints from baseline to 48 h. We selected pain reduction at 10 to 30 min as the most important in children in acute pain. No studies measured ease of or time to administration.

We have used GRADE narrative statements below to present the review findings [41]. For numerical results, see Table 2: Summary of findings table.

- 1) Comparing intranasal to intravenous analgesia

Heterogeneity of population, methods and study drugs precluded meta-analysis. GRADE assessments were performed for each individual study.

Pain relief: There may be little or no difference between

- single dose intranasal fentanyl and intravenous morphine for acute fracture 10 and 30 min after [29] (low certainty evidence);
- intranasal and intravenous fentanyl for post-operative pain at 10 and 15 min (though higher doses intranasal fentanyl were required) [30] (low certainty evidence);
- or intranasal and intravenous ketorolac for migraine headache [31] (low certainty evidence).

Rescue medication: There may be little or no difference between

- intranasal fentanyl or intravenous morphine for acute fractures [29] (low certainty evidence);
- or intranasal and intravenous ketorolac for migraine headache [31] (low certainty evidence).

Rescue medication was not reported in comparisons of intranasal and intravenous fentanyl in post-operative pain [30].

Adverse events: There may be little or no difference in between:

- intranasal fentanyl and intravenous morphine for acute fractures [29] (low certainty evidence);
- intranasal or intravenous fentanyl for post-operative pain [30] (low certainty evidence);
- or intranasal and intravenous ketorolac for migraine headache [31] (low certainty evidence).

No severe adverse events were recorded.

Acceptability: There may be little or no difference between intranasal and intravenous ketorolac for migraine headache [31] (low certainty evidence). Acceptability was not measured for the other comparisons in this group.

- 2) Comparing intranasal to intramuscular analgesia

For pain outcomes, poor reporting and missing data precluded meta-analysis. However, the studies had similar populations and study drugs, and a GRADE assessment was performed across the narrative synthesis of the studies.

Pain relief: There is probably little or no difference between fentanyl and diamorphine at 10 and 30 min, as those receiving intramuscular morphine (moderate certainty evidence). Any difference is unlikely to be clinically relevant [32–34].

Rescue medication: There may be no or little difference between patients receiving intranasal or intramuscular analgesia (low certainty evidence) (Additional file 6) [32–34].

Heterogeneous reporting precluded meta-analysis of adverse events and acceptability. However, the studies had similar populations and study drugs, and a GRADE assessment was performed across the narrative syntheses of the studies.

Adverse events: There may be little or no difference between intranasal fentanyl or diamorphine, and intramuscular morphine, though local manifestations differed somewhat. No severe adverse events were recorded (low certainty evidence) [32–34].

Acceptability:

- Children are less likely to be uncooperative or have a negative reaction to intranasal analgesia, than intramuscular morphine [32, 33] (high certainty evidence);
- providers find intranasal diamorphine more acceptable than intramuscular morphine [32] (high certainty evidence);
- Children and parents probably find intranasal diamorphine more acceptable than intramuscular morphine [32] (moderate certainty evidence).

3) Comparing different intranasal agents

Similarities between studies of intranasal ketamine compared to intranasal fentanyl allowed meta-analysis for this comparison, but not for intranasal fentanyl vs. placebo or standard vs. high concentration intranasal fentanyl.

**Fig. 3:**
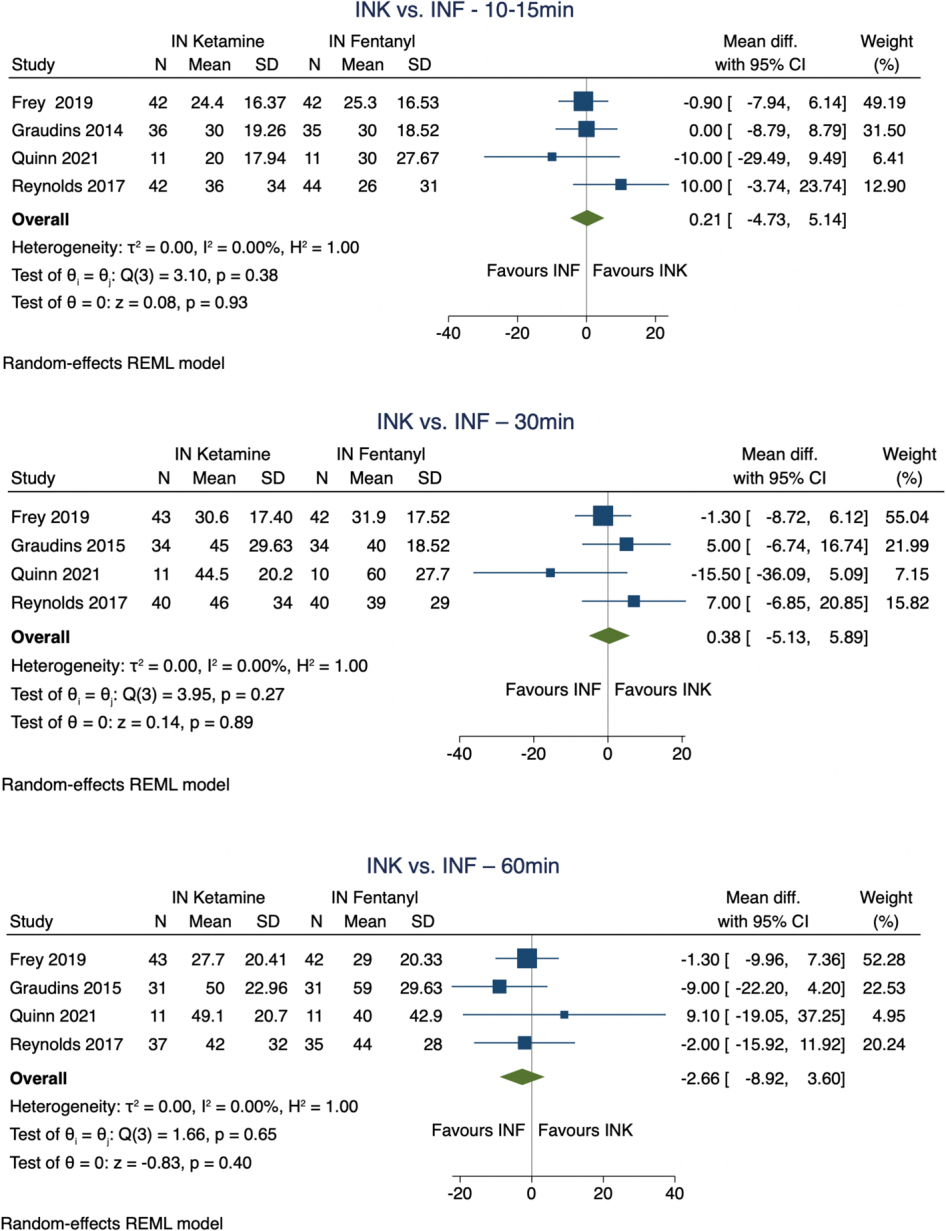
Meta-analysis – Pain reduction IN ketamine vs. IN fentanyl

Pain relief:

- There is little or no difference between intranasal ketamine and fentanyl at 10-15 min or 30 min after administration (high certainty evidence) [36, 37, 39, 40] (Fig. 3: Meta-analysis of pain relief from different intranasal agents). Asymmetric IQRs in Graudins 2015 led us to conduct a sensitivity analysis demonstrating that this study did not skew the overall results. (Additional file 2)
- There may be little or no difference in pain relief between intranasal fentanyl and placebo at 10 or 30 min compared to placebo [38] (low certainty evidence)*;* or standard (50mcg/ml) and high (300mcg/ml) concentration intranasal fentanyl at 10 and 30 min [35] (low certainty evidence).

Rescue medication:

- There is probably little or no difference between intranasal ketamine and intranasal fentanyl [36, 37, 39, 40] (moderate certainty evidence) (Additional file 7).
- Children receiving standard concentration intranasal fentanyl may require rescue analgesia more often than children receiving high concentration [35].

Rescue medication was not reported in comparisons of intranasal fentanyl and placebo.

Adverse events:

- Patients receiving intranasal ketamine are at higher risk of adverse events than those receiving intranasal fentanyl, though these are non-severe (Additional file 8, 9); and at a higher risk of sedation, though the degree of sedation was light (high certainty evidence) (Additional file 10) [36, 37, 39, 40]. We assumed “sleepiness” and “drowsiness” in Reynolds 2017 corresponded to sedation in the other studies. A sensitivity analysis did not find Reynolds 2017 to alter the results (Additional file 3).
- There may be little or no difference between intranasal fentanyl or placebo, or standard or high concentration intranasal fentanyl (low certainty evidence) [35, 38].

Acceptability: There may be little or no difference between intranasal ketamine and intranasal fentanyl (low certainty evidence) [36]. Acceptability was not assessed for the other comparisons of intranasal agents.

## Discussion

General interpretation in context of other evidence.

Our review took a broad approach, assessing effects of intranasal analgesia in children with acute moderate to severe pain of any cause; assessing their effect on pain, risk of adverse events, use of rescue analgesia, acceptability/tolerability, and ease and speed of administration. Unlike previous reviews, our review was not restricted to a single drug, setting or aetiology; although we did exclude studies assessing pain prevention and aetiology-specific treatments (such as triptans for migraines, or procedural analgesia).

Two previous systematic reviews have explored similar questions. Murphy 2014 assessed the use of intranasal fentanyl in acute pain in children; including three of the same trials – Borland 2007, Borland 2011 and Younge 1999. The review included one small study per comparison, but still concluded that pain reduction with intranasal fentanyl was equivalent to intravenous morphine (high level of certainty); intranasal fentanyl being superior to intramuscular morphine at 10 min; and that respiratory, circulatory or GCS depression, and use of rescue medication were similar (moderate certainty). Though we come to similar conclusions, our assessment of certainty is lower, downgraded for imprecision, with few studies and a low number of participants.

Silva et al. 2020 reviewed the use of intranasal ketamine compared to intranasal fentanyl in the management of acute pain in children in the emergency department. The review included the same four studies for this comparison that we included in our review, coming to conclusions similar to ours regarding pain relief, adverse events sedation and acceptability.

The studies included in this review that have not been included in previous systematic reviews add information on intranasal diamorphine compared to intramuscular morphine [32, 34], intranasal fentanyl to intranasal fentanyl [30], intranasal fentanyl to intranasal saline [38], and intranasal ketorolac to intravenous ketorolac [31]. The addition of these studies would not have changed the results of previous reviews, but add information on the overall efficacy and use of intranasal analgesia compared to other parenteral routes of administration.

### Limitations of included evidence

All included studies included pain as an outcome, with all but one as the primary outcome. However, the study drugs, pain scores and level of reporting varied. This heterogeneity made meta-analysis impractical or impossible for most comparisons and outcomes. The overall heterogeneity and paucity of evidence resulted in few moderate or high certainty conclusions. Furthermore, means and medians of continuous outcomes are not alone ideal for assessing pain relief. Combining continuous outcomes and threshold values of clinical significance with dichotomous outcomes of pain relief and pain freedom are likely more clinically useful [28].

Though we did not attempt to find dosages or dosing regimens, we noted that the different studies employed varied dosing regimens. Intranasal fentanyl was dosed at 1.0mcg/kg to 2mcg/kg, intranasal diamorphine at 0.1 mg/kg, and intranasal ketamine at 1 mg/kg to 1.5 mg/kg; and the number of additional doses varied from zero to “as needed”. The optimal dose and administration regime remains uncertain. Overall, it seems reasonable to assume that intranasal analgesics may perform worse than intravenous analgesics of the same potency (i.e. intranasal fentanyl vs. intravenous fentanyl, intranasal ketorolac vs. intravenous ketorolac), but better than intravenous analgesics of lower potency (i.e. intranasal fentanyl vs. intravenous morphine).

All studies reported adverse events, though there was again significant heterogeneity in how they were reported. While some studies provided detailed lists of all adverse events, others only described that there were no recorded differences. Overall, no severe adverse events of deep sedation, respiratory depression or circulatory depression were reported in any of the included studies, with over a thousand children receiving opioids or ketamine. This suggests that these events are uncommon, irrespective of agent or route, and any difference is unlikely to be uncovered in the relatively small RCTs.

Sedation did not appear to be different between routes of administration. However, between intranasal agents, ketamine had a higher risk of sedation than fentanyl. Though initially considered an adverse event, none of the cases of sedation were deep. As such, ketamine may be preferred in situations where light sedation may be desired.

Few studies explored acceptability or tolerance, and none ease or speed of administration. As these are common rationales for intranasal analgesia, this was surprising. The difference between the large effect sizes for acceptability and related outcomes in the intranasal/intramuscular-studies, and the lack of a difference in the intranasal/intravenous-study is stark, but may be explained by study medication, pain aetiology, or participant age. The study by Tsze 2022 was the only one to include children with migraine headache and used an NSAID as analgesic [31]. With a mean age of 15 years, this study also included an older children compared to other trials. It is possible that younger children overall find intramuscular/intravenous administration less acceptable, and that acceptability of intranasal may be higher with opioids than NSAIDs.

Costs and cost-effectiveness were not assessed in this review but should be considered in further research.

### Limitations of the review

Nine of eleven authors failed to answer our requests for further information. We included these studies, but the lack of information may have influenced our conclusions. Additionally, two trials were excluded after initial inclusion – one during extraction due to the use of pathology-specific treatments [42], and the other during analysis because it was a study of pain prevention [43]. The exclusion of these studies had no impact on our final conclusions. Other departures from protocol (Additional file 11) are unlikely to have affected our analyses or conclusions. We only included English-language studies, and may have missed trials in other languages. Furthermore, no subgroup analyses were conducted in this review, due to lack of patient level data.

### Implications for practice and future research

Intranasal administration of high potency opioids probably gives pain relief equivalent to intramuscular morphine, with similar adverse events; and children, parents, and providers prefer analgesia by the intranasal route. Intranasal analgesia may be considered instead of intramuscular for children with acute moderate to severe pain.

Intranasal ketamine gives pain relief equivalent to intranasal fentanyl, but with a higher rate of mild adverse events and sedation. Whether or not sedation is desired should inform the choice of agent.

The studies included in those review are mostly small, and further research should aim to.

- replicate studies of intranasal analgesia vs. IV analgesia, with more participants and for different causes of pain;
- explore the efficacy of intranasal analgesia in children with different aetiologies of pain, along with dosages;
- assess time to analgesic administration, as well as ease of administration and actual time to analgesia from identified pain;
- use larger datasets to assess uncommon severe adverse events.

## Conclusions

Our review suggests that intranasal analgesics can be considered as an alternative to intramuscular analgesics in children with acute moderate to severe pain; and may be an alternative to intravenous administration. Intranasal ketamine gives similar pain relief as fentanyl, but causes more sedation, which should inform the choice of intranasal agent.

### Supplementary Information

**Additional file1.** Search strategy.

**Additional file 2.** Sensitivity analysis – Pain – INK vs INF.

**Additional file 3.** Sensitivity analysis – Sedation – INK vs INF.

**Additional file 4.** Funnel plot – Pain – INK vs INF.

**Additional file 5.** Ongoing and/or unfinished studies.

**Additional file 6.** Meta-analyses for other rescue medication.

**Additional file 7.** Meta-analysis for Rescue medication – INK vs INF.

**Additional file 8.** Meta-analysis for adverse events – INK vs INF, total.

**Additional file 9.** Meta-analysis for adverse events – INK vs INF, specific events.

**Additional file 10.** Meta-analysis for sedation – INK vs INF.

**Additional file 11.** Departures from protocol.

## Data Availability

All included studies are available in their respective journals, with all supporting data clearly cited in the manusscript. A comprehensive list of screened references can be supplied on demand. Additional analyses are available in the supplements or upon demand.

## Abbreviations

IV: Intravenous
IM: Intramuscular
IN: Intranasal
RCT: Randomised controlled trial/Randomised clinical trial
VAS: Visual Analogue Scale
ROB 2.0: Risk of Bias 2.0
GRADE: Grading of Recommendations, Assessment, Development and Evaluations
MD: Mean Difference
95% CI: 95% Confidence Interval
SD: Standard Deviation
IQR: Interquartile Range
INK: Intranasal Ketamine
INF: Intranasal Fentanyl
NaCl: Sodium-Chloride
FPS-R: Faces Pain Scale-Revised
WBFPS: Wong Baker Faces Pain Scale
NRS: Numeric Rating Scale
UMSS: University of Michigan Sedation Scale
