# Supplementary material for: Intranasal analgesia for acute moderate to severe pain in children – a systematic review and meta-analysis
Source: BMC Pediatr. 2023 Aug 18;23:405. doi: 10.1186/s12887-023-04203-x (PMC10436645; doi:10.1186/s12887-023-04203-x)
Supplement: Supplementary file 1 — Additional file1. Search strategy. [file 12887_2023_4203_MOESM1_ESM.docx]

**Search strategy example for PROSPERO adopted for Ovid Embase.**

1. exp intranasal drug administration/
2. exp nose spray/
3. exp nose mucosa/
4. exp nose cavity/
5. intranasal*.ti,ab,kw.
6. transnasal*.ti,ab,kw.
7. nasal*.ti,ab,kw.
8. nostril*.ti,ab,kw.
9. transmucosal*.ti,ab,kw.
10. or/1-9 [Concept#1: Intranasal]
11. exp analgesia/
12. exp analgesic agent/
13. analgesi*.ti,ab,kw.
14. analgetic*.ti,ab,kw.
15. 15  analgesic*.ti,ab,kw.
16. oligoanalgesia.ti,ab,kw.
17. (relief* adj2 pain).ti,ab,kw.
18. exp opiate/
19. exp opiate derivative/
20. exp morphine derivative/
21. exp morphine/
22. (morphine or morphia or contin or oramorph or duramorph or morphinene or epimorph or miro or morfin or morfine or morphin or morphinium or morphium or opso or skenan).ti,ab,kw.
23. exp hydromorphone/
24. (biomorphyl or cofalaudid or dihydromorphinone or dihydromorphone or diladid or dilaudid or dimorphone or dolonovag or exalgo or hydal or hydromorph or hydromorphinone or hydromorphone or hydromorphone or hydrostat or hymorphan or jurnista or laudaconumor novolaudon or opidol or paliadon or palladon or palladone or rexaphon or semcoxor sophidone).ti,ab,kw.
25. exp dihydromorphine/
26. (dihydromorphine or paramorfan or paramorphan or methylmorphinan or hydromorphine).ti,ab,kw.
27. exp diamorphine/
28. (diamorphine or diacetylmorphine or diagesil or diamorf or heroin or acetomorphine or diacephine or diaphorin or heroine or morphacetin).ti,ab,kw.
29. exp buprenorphine/
30. exp buprenorphine plus naloxone/
31. (acimaphin or acinorphin or addictex or addnok or algesalona or anorfin or astec or belbuca or brixadi or bugnanto or bunondol or bunorfin or bunov or bupacal or bupainx or bupalster or bupan or bupeaze or bupensan or buphin or buplab or buplast or bupramyl or bupre-1 or bupre-hexal or buprefarm or bupremyl or buprenaddict or buprenal or buprenex or buprenocan or buprenoratiopharm or buprenorfin or buprenorfina or buprenorphine or buprenorphine or buprenorphine or bupretec or buprex or buprine or busette or busiete or butec or butrans or buvera or buvidal or carlosafine or dolotec or durlevatec or feliben or finibron or hapoctasin or laribon or lepetan or melody or mitoren or molterfin or natzon or nimedol or noprex or norfinox or norphin or norspan or norvipren or panitaz or pentorel or prefibin or prefin or prenorvine or prenotrix or probuphine or ramatrix or ravata or reletrans or relevtec or sevodyne or sixmo or somnena or sublocade or subutex or temgesic or trephine or thorbup or transtec or tranzileve or triquisic or suboxone).ti,ab,kw.
32. exp codeine/
33. (ardinex or codein or codeine or codicaps or codipertussin or codyl or Isocodeine or methyl morfine or methyl morphine or methylmorfine or methylmorphine or "morphine methyl ether" or "morphine monomethyl ether" or pentuss or tussicalm).ti,ab,kw.
34. exp methadone/
35. exp methadone plus naloxone/
36. (adanon or adanon or algolysin or algoxale or althose or amidon or amidona or amidone or amidosan or anadon or biodone or butalgin or deamin or depridol or diaminon or dianone or dolafin or dolamid or dolesone or dolmed or dolophine or dorex or dorexol or eptadone or fenadon or gobbidona or heptadon or heptanon or ketalgin or mecodin or mepecton or mephenon or metadol or metadon or metasedin or methaddict or methadone or methadone or methadose or methaforte or methex or miadone or moheptan or pallidone or phenadon or phymat or physeptone or physeptone or pinadone or polamidon or polamivet or polamivit or sinalgin or symoron or westadone).ti,ab,kw.
37. exp fentanyl/
38. (fentanyl or phentanyl or fentanest or sublimaze or duragesic or durogesic or fentora or epufen or fentalis or fentamat or fentanex or instanyl or leptanal or mezolar or pecfent or rapinyl or recuvyra or subsys or tanyl or tilotrans or transfenta).ti,ab,kw.
39. exp ketamine/
40. (ketamine or ketalar or ketaset or ketanest or calipsol or kalipsol or calypso or cyclohexanone or methylaminocyclohexanone or anesject or imalgene or kalipsol or ketamine or keta-hameln or ketaject or ketalin or ketamax or ketanest or ketased or ketaset or ketaved or ketavet or ketmin or ketoject or ketolar or narkamon or narketan or soon-soon or tekam or velonarcon or vetalar).ti,ab,kw.
41. exp hydrocodone/
42. (hydrocodone or hydrocodone or dihydrocodeinone or dicodid or robidone or hydrate or hydrocodeinonebitartrate or hydrocon or codinovo or hycodan or hycon or bekadid or dico or dihydrocodone or hydrocodonum).ti,ab,kw.
43. exp hydromorphone/
44. exp hydromorphone plus naloxone/
45. (hydromorphone or dihydromorphinone or biomorphyl or cofalaudid or dihydromorphone or diladid or dilaudid or dimorphone or dolonovag or exalgo or hydal or hydromorph or hydromorphinone or hydrochloride or hydromorphone or hydromorphone or hydrochloride or hydrostat or hymorphan or jurnista or laudaconum or novolaudon or opidol or paliadon or retardkaps or palladon or palladone or rexaphon or semcox or sophidone or sophidone).ti,ab,kw.
46. exp oxycodone/
47. (oxycodone or hydroxydihydrocodeinone or abtard or accordeon or alivio or bionine or bionone or bolodorm or broncodal or bucodal or cafacodal or candox or cardanon or carenoxal or carexil or codenon or codilek or codex or codoxy or oxycodone or terephthalate or contiroxil or daloxy or dancex or dihydrohydroxycodeinone or dihydrone or dinarkon or dolanor or dolocodon or dyxal or endone or eubine or eucodal or eucodale or eucodalum or eudin or eukdin ro eukodal or eumorphal or eurodamine or eutagen or hydrocodal or hydroxycodeinoma or ixyldone or lenocod or leveraxo or longtec or ludonal or lynlor or medicodal or narcobasina or narcobasine or narcosin or nargenol or narodal or nucodan or oksikodon or olbete or onexila or opton or orionox or ossicodone or oxanest or oxaydo or oxecta or oxeltra or oxicodona or oxicone or oxicontin or oxiconum or oxidol or oxidolor or oxikodon or oxikon or oxpian or oxycan or oxycod or oxycodeinonhydrochloride or oxycodone or oxycodone or oxycodon or oxycodone or oxycodonhydrochlorid or oxycodyl or oxycone or oxyconica or oxyconicur or oxyconoica or oxycontin or oxydol or oxydolor or oxydon or oxydose or oxyfast or oxygerolan or oxygesic or oxyir or oxykodon or oxykon or oxylor or oxynorm or oxynormoro or oxypro or oxyratio or oxytina or pancodine or pavinal or percolone or pronarcin or reltebon or remoxy or renocontin or roxicodone or roxybond or roxycodone or shortec or sinthiodal or stupenal or supeudol or taioma or tebodal or tekodin or thecodin or xancodal or xtampa or xtampza or zarenoxin or zomestine).ti,ab,kw.
48. exp ketobemidone/
49. (cetobemidone or ciba or cliradon or cliradone or cymidon or ketobemidone).ti,ab,kw.
50. exp pethidine/
51. (algil or alodan or centralgin or centralgine or cluyer or demero or demerol or dispadol or dolanquifa or dolantal or dolantin or dolantina or dolantine or dolargan or dolcontral or dolenal or dolestin or dolestine or dolin or dolocontral or doloneurin or doloneurotrat or dolor or dolosa or dolosal or dolosan or dolsin or dolvanol or endolate or isonipecaine or pethidine or lidol or lydol or mefedina or mepadin or meperdol or meperiden or meperidine or meperidine or meperidol or mephedine or meperidine or methylphenylcarbethoxypiperidine or mialgin or neomochin or operidine or opistan or pantalgin or petadin or petantin or petantina or pethanol or pethidine or pethidine or pethidine or petidin or phetidine or piridosal or sauteralgyl or simesalgina or supplosal or synlaudine).ti,ab,kw.
52. exp tapentadol/
53. (aspadol or nucynta or palexia or tapenta or tapentadol or tapal or yantil or yantuk).ti,ab,kw.
54. or/11-53 [Concept#2: Analgesia/ Analgesic agents]
55. 10 and 54 [Concept #1 and #2 combined]
56. random*.tw. or placebo*.mp. or double-blind*.tw. [Filter for Randomized controlled trials]
57. (exp animal/ or exp juvenile animal/ or adult animal/ or animal cell/ or 57 animal tissue/ or nonhuman/ or animal experiment/ or animal model/) not human/ [Filter for Randomized controlled trials]
58. (55 and 56) not 57 [Concept #1 and #1 combined and filtered for RCT- studies and non-human studies applied]
