# Supplementary material for: Intranasal analgesia for acute moderate to severe pain in children – a systematic review and meta-analysis
Source: BMC Pediatr. 2023 Aug 18;23:405. doi: 10.1186/s12887-023-04203-x (PMC10436645; doi:10.1186/s12887-023-04203-x)
Supplement: Supplementary file 2 — Additional file 2. Sensitivity analysis – Pain – INK vs INF. [file 12887_2023_4203_MOESM2_ESM.pdf]

Sensitivity analysis – Pain INF vs. INK excl. Graudins

	Treatment			Control			Hedges's g	Weight
Study	N	Mean	SD	N	Mean	SD	with 95% CI	(%)
**10**								
Frey	42	24.4	16.367177	42	25.3	16.532502	-0.05 [-0.48, 0.37]	12.71
Quinn	11	30	14.709304	11	50	25.7	-0.92 [-1.77, -0.07]	3.17
Reynolds	42	36	34	44	26	31	0.30 [-0.12, 0.73]	12.86
Heterogeneity: $\tau^2 = 0.22$, $I^2 = 75.80\%$, $H^2 = 4.13$							-0.13 [-0.75, 0.49]	
Test of $\theta_i = \theta_j$: Q(2) = 6.57, p = 0.04								
**20**								
Quinn	11	43.63	20.63	11	57.27	29.36	-0.52 [-1.34, 0.30]	3.41
Reynolds	39	44	36	44	35	29	0.27 [-0.15, 0.70]	12.41
Heterogeneity: $\tau^2 = 0.20$, $I^2 = 64.54\%$, $H^2 = 2.82$							-0.04 [-0.80, 0.72]	
Test of $\theta_i = \theta_j$: Q(1) = 2.82, p = 0.09								
**30**								
Frey	43	30.6	17.397286	42	31.9	17.524452	-0.07 [-0.50, 0.35]	12.86
Quinn	11	44.55	20.18	10	59.09	27.73	-0.58 [-1.42, 0.26]	3.23
Reynolds	40	46	34	40	39	29	0.22 [-0.22, 0.65]	12.05
Heterogeneity: $\tau^2 = 0.02$, $I^2 = 17.75\%$, $H^2 = 1.22$							-0.02 [-0.34, 0.30]	
Test of $\theta_i = \theta_j$: Q(2) = 2.92, p = 0.23								
**60**								
Frey	43	27.7	20.408355	42	29	20.334977	-0.06 [-0.48, 0.36]	12.86
Quinn	11	49.09	20.71	11	41.81	42.85	0.21 [-0.60, 1.01]	3.51
Reynolds	37	42	32	35	44	28	-0.07 [-0.52, 0.39]	10.92
Heterogeneity: $\tau^2 = 0.00$, $I^2 = 0.00\%$, $H^2 = 1.00$							-0.03 [-0.32, 0.26]	
Test of $\theta_i = \theta_j$: Q(2) = 0.38, p = 0.83								
Heterogeneity: $\tau^2 = 0.00$, $I^2 = 0.00\%$, $H^2 = 1.00$								
Test of $\theta_i = \theta_j$: Q(10) = 13.01, p = 0.22								
Test of group differences: $Q_b(3) = 0.10$, p = 0.99								

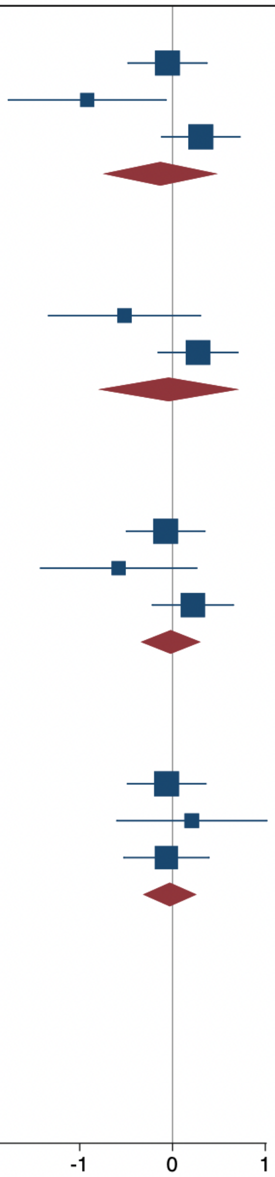

Random-effects REML model

Sensitivity analysis – Pain INK vs. INF incl. Graudins

Study	Treatment N	Treatment Mean	Treatment SD	Control N	Control Mean	Control SD	Hedges's g with 95% CI	Weight (%)
**10**								
Frey 2019	42	24.4	16.367177	42	25.3	16.532502	-0.05 [-0.48, 0.37]	9.74
Graudins 2015	36	30	19.259259	35	30	18.518519	0.00 [-0.46, 0.46]	8.26
Quinn 2021	11	30	14.709304	11	50	25.7	-0.92 [-1.77, -0.07]	2.43
Reynolds 2017	42	36	34	44	26	31	0.30 [-0.12, 0.73]	9.85
Heterogeneity: τ^2 = 0.06, I^2 = 50.39%, H^2 = 2.02							-0.05 [-0.40, 0.31]	
Test of $\theta_i = \theta_j$: Q(3) = 6.57, p = 0.09								
**20**								
Quinn 2021	11	43.63	20.63	11	57.27	29.36	-0.52 [-1.34, 0.30]	2.61
Reynolds 2017	39	44	36	44	35	29	0.27 [-0.15, 0.70]	9.50
Heterogeneity: τ^2 = 0.20, I^2 = 64.54%, H^2 = 2.82							-0.04 [-0.80, 0.72]	
Test of $\theta_i = \theta_j$: Q(1) = 2.82, p = 0.09								
**30**								
Frey 2019	43	30.6	17.397286	42	31.9	17.524452	-0.07 [-0.50, 0.35]	9.85
Graudins 2015	34	45	29.62963	34	40	18.518518	0.20 [-0.27, 0.67]	7.88
Quinn 2021	11	44.55	20.18	10	59.09	27.73	-0.58 [-1.42, 0.26]	2.48
Reynolds 2017	40	46	34	40	39	29	0.22 [-0.22, 0.65]	9.23
Heterogeneity: τ^2 = 0.00, I^2 = 0.00%, H^2 = 1.00							0.05 [-0.19, 0.29]	
Test of $\theta_i = \theta_j$: Q(3) = 3.46, p = 0.33								
**60**								
Frey 2019	43	27.7	20.408355	42	29	20.334977	-0.06 [-0.48, 0.36]	9.85
Graudins 2015	31	50	22.962963	31	50	29.62963	0.00 [-0.49, 0.49]	7.24
Quinn 2021	11	49.09	20.71	11	41.81	42.85	0.21 [-0.60, 1.01]	2.69
Reynolds 2017	37	42	32	35	44	28	-0.07 [-0.52, 0.39]	8.37
Heterogeneity: τ^2 = 0.00, I^2 = 0.00%, H^2 = 1.00							-0.02 [-0.27, 0.23]	
Test of $\theta_i = \theta_j$: Q(3) = 0.39, p = 0.94								

Heterogeneity: τ^2 = 0.00, I^2 = 0.00%, H^2 = 1.00

Test of $\theta_i = \theta_j$: Q(13) = 13.60, p = 0.40

Test of group differences: $Q_b(3)$ = 0.26, p = 0.97

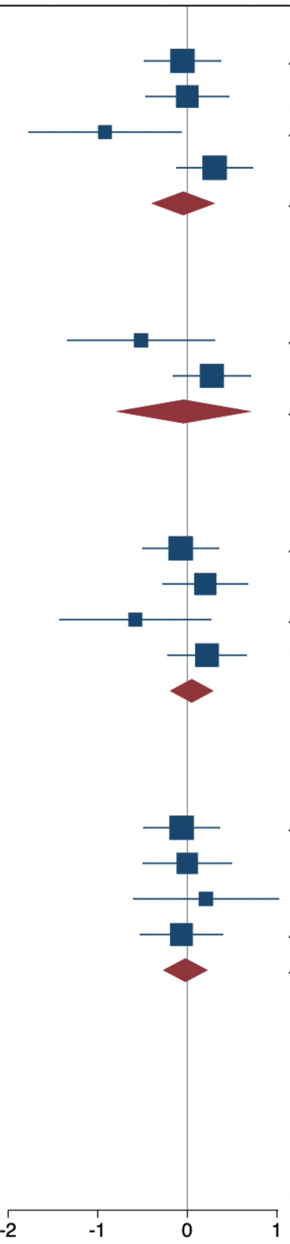

Random-effects REML model
