# Supplementary material for: Intranasal analgesia for acute moderate to severe pain in children – a systematic review and meta-analysis
Source: BMC Pediatr. 2023 Aug 18;23:405. doi: 10.1186/s12887-023-04203-x (PMC10436645; doi:10.1186/s12887-023-04203-x)

Sensitivity analysis – Sedation INK vs. INF excl. Reynolds

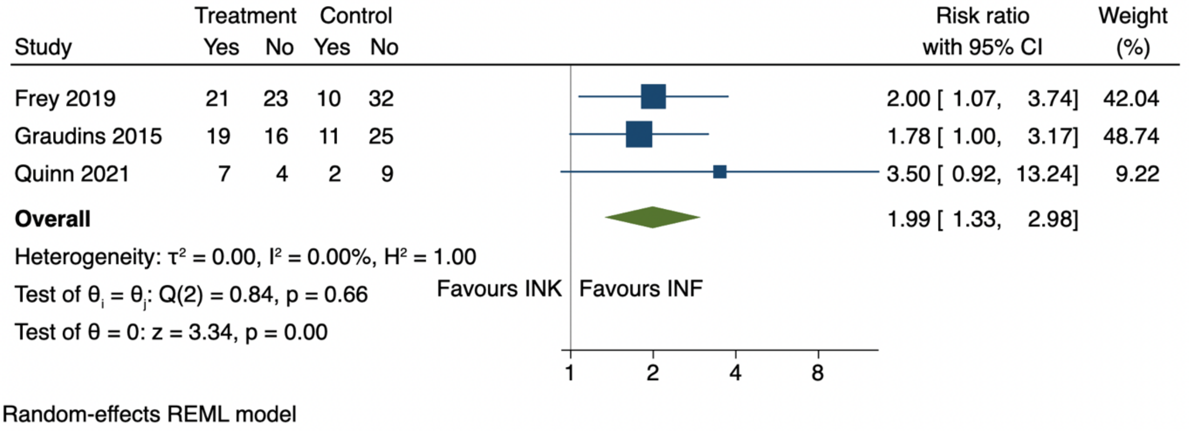

Sensitivity analysis - Sedation INK vs. INF incl. Reynolds

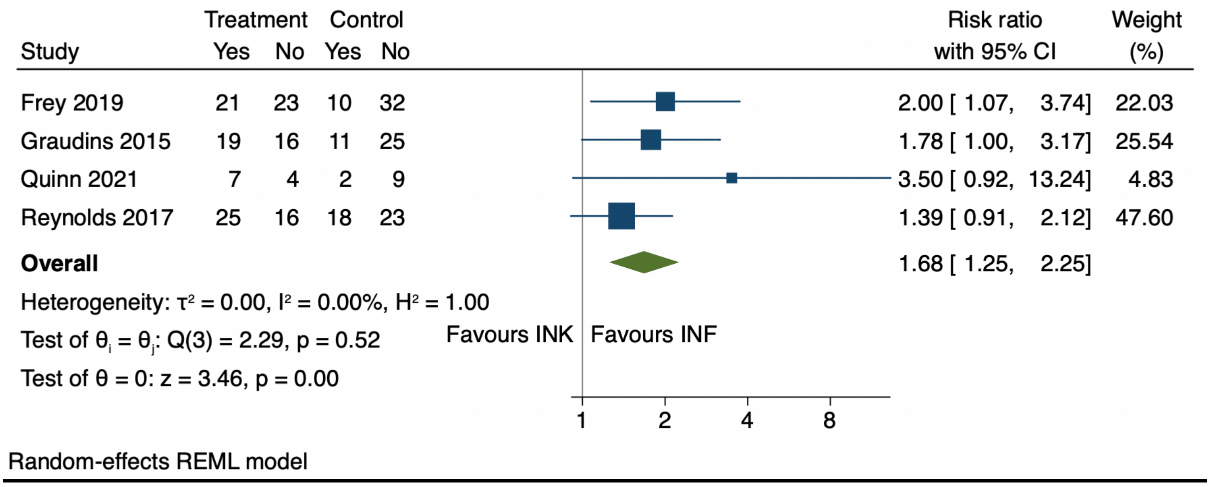

Supplement: Supplementary file 3 — Additional file 3. Sensitivity analysis – Sedation – INK vs INF. [file 12887_2023_4203_MOESM3_ESM.pdf]
