# Supplementary figures and images for: Intranasal analgesia for acute moderate to severe pain in children – a systematic review and meta-analysis
Source: BMC Pediatr. 2023 Aug 18;23:405. doi: 10.1186/s12887-023-04203-x (PMC10436645; doi:10.1186/s12887-023-04203-x)

## Funnel plot - Pain - INK vs. INF

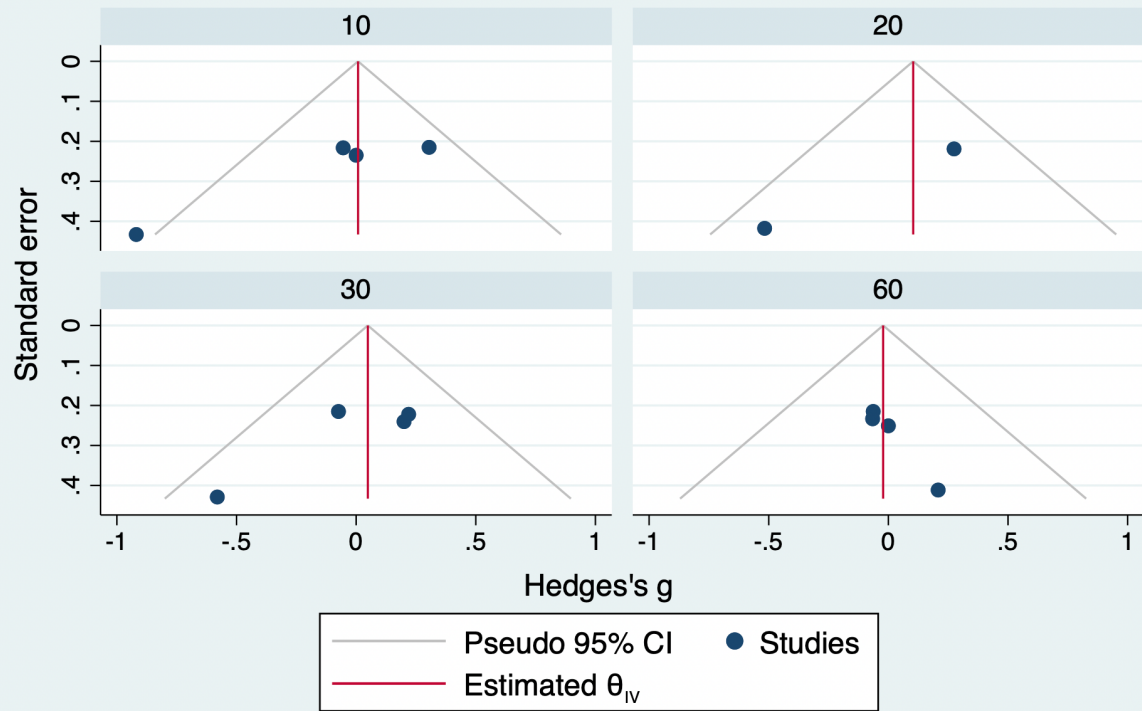

Graphs by Time

Supplement: Supplementary file 4 — Additional file 4. Funnel plot – Pain – INK vs INF. [file 12887_2023_4203_MOESM4_ESM.pdf]

## Other - Rescue medications

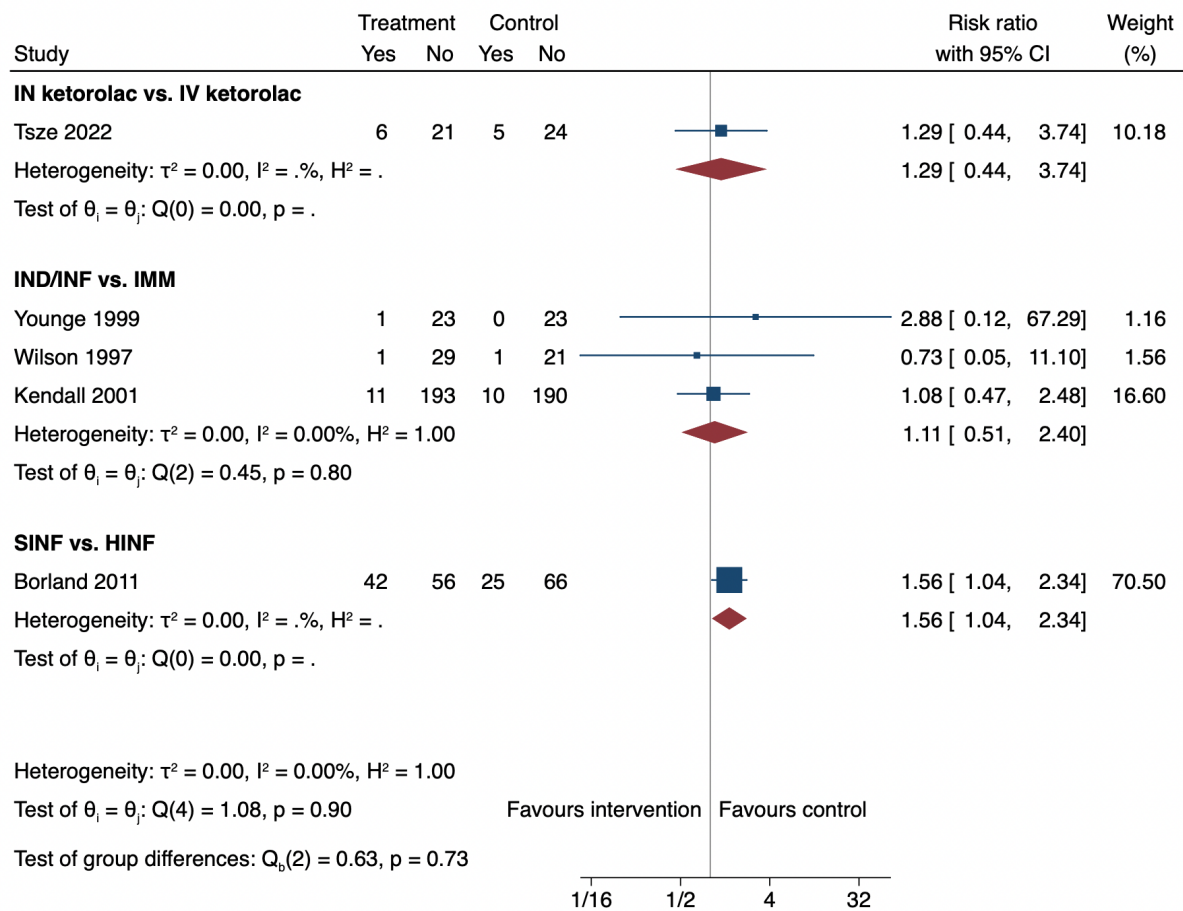

Random-effects REML model

Supplement: Supplementary file 6 — Additional file 6. Meta-analyses for other rescue medication. [file 12887_2023_4203_MOESM6_ESM.pdf]

# INK vs. INF – Rescue medications

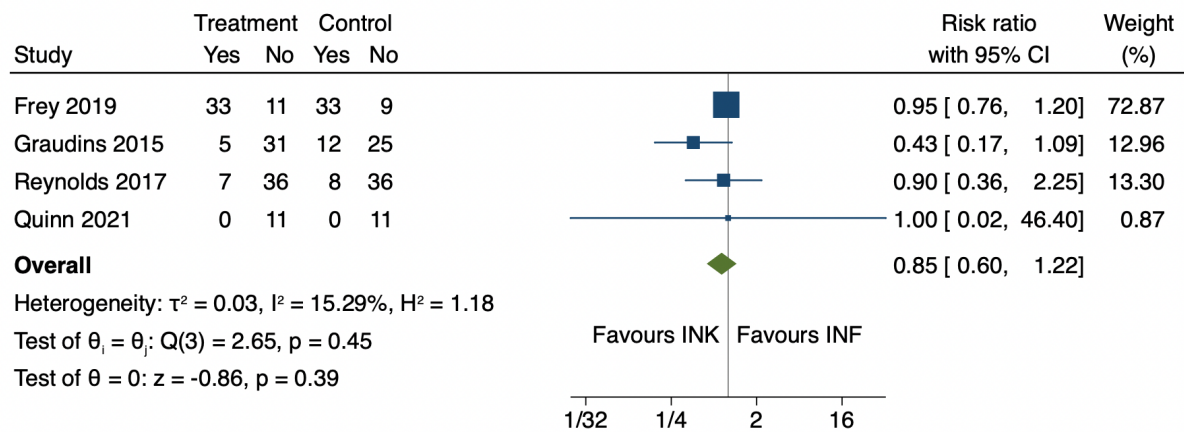

Random-effects REML model

Supplement: Supplementary file 7 — Additional file 7. Meta-analysis for Rescue medication – INK vs INF. [file 12887_2023_4203_MOESM7_ESM.pdf]

INK vs. INF – Adverse events total

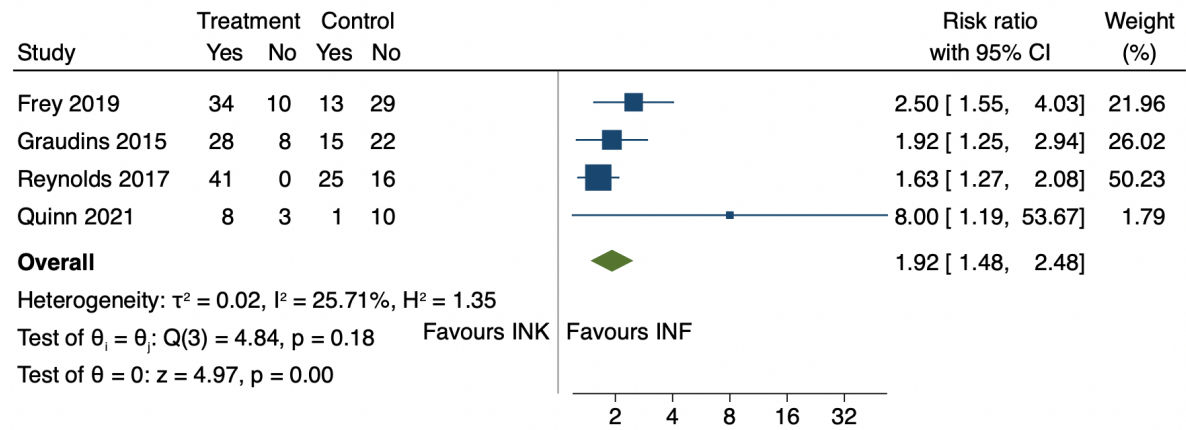

Random-effects REML model

Supplement: Supplementary file 8 — Additional file 8. Meta-analysis for adverse events – INK vs INF, total. [file 12887_2023_4203_MOESM8_ESM.pdf]

# INK vs. INF – Adverse events selected

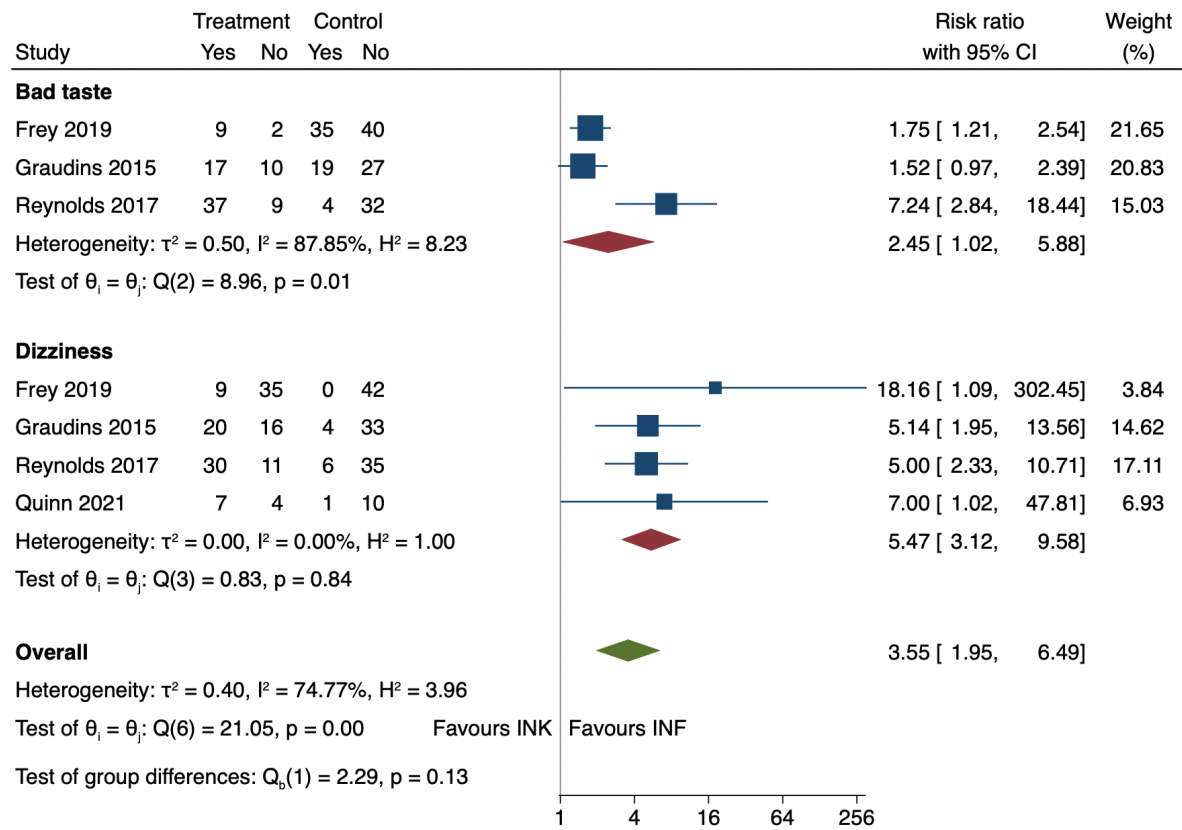

Random-effects REML model

Supplement: Supplementary file 9 — Additional file 9. Meta-analysis for adverse events – INK vs INF, specific events. [file 12887_2023_4203_MOESM9_ESM.pdf]

# INK vs. INF – Sedation

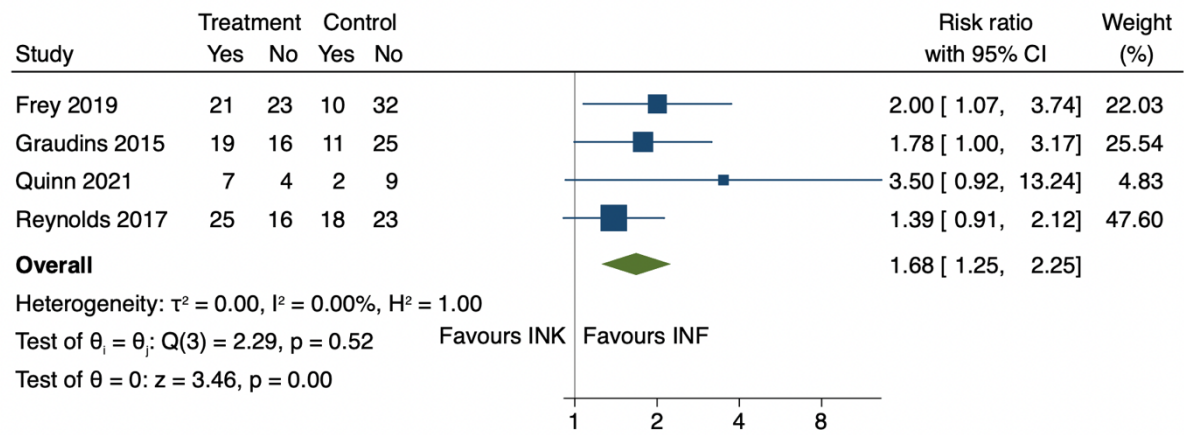

Random-effects REML model

Supplement: Supplementary file 10 — Additional file 10. Meta-analysis for sedation – INK vs INF. [file 12887_2023_4203_MOESM10_ESM.pdf]
