# Supplementary material for: Intranasal analgesia for acute moderate to severe pain in children – a systematic review and meta-analysis
Source: BMC Pediatr. 2023 Aug 18;23:405. doi: 10.1186/s12887-023-04203-x (PMC10436645; doi:10.1186/s12887-023-04203-x)
Supplement: Supplementary file 5 — Additional file 5. Ongoing and/or unfinished studies. [file 12887_2023_4203_MOESM5_ESM.pdf]

Ongoing studies – intranasal analgesia for acute pain in children	
Identifier	Description
NCT02985177	A RCT of a combination of analgesics for pain management in children with a suspected fracture (CAST).<br>Status: Unknown. Not yet recruiting September 20th 2018.<br>INF + Oral hydromorphone vs. INF + Oral ibuprofen.
NCT02573714	Sub dissociative Intranasal ketamine for pediatric sickle cell pain crises<br>Status: unknown. Recruiting March 14th 2019.<br>INK vs. Standard pain therapy.
NCT00882960	Intranasal Fentanyl for Pain Management<br>Status: unknown. Recruiting May 4th 2011.<br>INF vs. IVF.
NCT05057689	Safety and efficacy of intranasal dexmedetomidine, fentanyl and midazolam in the pediatric emergency room.<br>Status: Not yet recruiting March 3rd 2022.<br>IN dexmedetomidine vs. INF vs. IN midazolam.
NCT03950817	Nebulized Sub-dissociative
IRCT20111114008104N15	Comparison of analgesic effects and morphine consumption of intravenous morphine sulfate and intranasal dexmedetomidine with intravenous morphine sulfate in patients with extremity fractures presented in emergency departments<br>Status: Recruitment complete January 2nd 2019.<br>IN Dexmedetomidine+IVM vs. IVM
