# Supplementary material for: Intranasal analgesia for acute moderate to severe pain in children – a systematic review and meta-analysis
Source: BMC Pediatr. 2023 Aug 18;23:405. doi: 10.1186/s12887-023-04203-x (PMC10436645; doi:10.1186/s12887-023-04203-x)
Supplement: Supplementary file 11 — Additional file 11. Departures from protocol. [file 12887_2023_4203_MOESM11_ESM.pdf]

Departures from protocol	
1) No Network Meta-analysis (NMA)	We initially planned on performing NMA where possible, but the inadequate reporiting of some results and overall paucity of evidence did not produce a network that was sufficiently robust for NMA
2) Revman	We abandoned using RevMan5 for practical reasons, as we had technical issues with the software. Instead we used multiple other programs, like Covidence, Microsoft Excel, Microsoft Word, STATA, The Campbell Collaboration Effect size calculator and MedCalc.org.
3) Partial use of Covidence.	We attempted using Covidence for data extraction and Risk of Bias assessment, in addition to the planned screening; but abandoned this as it did not sufficiently meet our needs. We instead used primarily Microsoft Excel for data extraction, and a ROB2.0 spreadsheet for risk of bias assessment.
4) PubMed alerts	We did not continuously apply PubMeds MyNCBI-alerts, instead relying on the final search.
5) Risk of Bias-tool	We did not use the Cochrane Risk of Bias assessment tool, but instead ROB2.0 for intervention reviews.
6) Pathology specific treatments	We excluded pathology specific treatments (such as triptans for migraine headache) after having started data extraction, as we found these would not answer our research questions.
7) Additional members	Ekaterina Spiridonova and Daniel Munblit joined the review team after publishing the protocol
8) Changes in reporting	We changed reporting of pain scales to better fit the recommended reporting standards from Busse 2015. This meant using mean differences where possible, with converted pain scores to 100mm/10cm VAS in metaanalyses. Changes in pain scores relative to degree of clinical significance was noted. SMDs were still calculated in addition to these.
9) Added exclusions criteria	Pain prevention added to exclusion criteria
